# Optimisation of CT protocols in PET-CT across different scanner models using different automatic exposure control methods and iterative reconstruction algorithms

**DOI:** 10.1186/s40658-021-00404-4

**Published:** 2021-07-31

**Authors:** Sarah-May Gould, Jane Mackewn, Sugama Chicklore, Gary J. R. Cook, Andrew Mallia, Lucy Pike

**Affiliations:** King’s College London & Guy’s and St Thomas’ PET Centre, School of Biomedical Engineering and Imaging Sciences, King’s College London, St Thomas’ Hospital, Westminster Bridge Road, London, SE1 7EH UK

**Keywords:** PET-CT, Automatic exposure control, Optimisation, Protocol matching, Iterative reconstruction

## Abstract

**Background:**

A significant proportion of the radiation dose from a PET-CT examination is dependent on the CT protocol, which should be optimised for clinical purposes. Matching protocols on different scanners within an imaging centre is important for the consistency of image quality and dose. This paper describes our experience translating low-dose CT protocols between scanner models utilising different automatic exposure control (AEC) methods and reconstruction algorithms.

**Methods:**

The scanners investigated were a newly installed Siemens Biograph mCT PET with 64-slice SOMATOM Definition AS CT using sinogram affirmed iterative reconstruction (SAFIRE) and two GE Discovery 710 PET scanners with 128-slice Optima 660 CT using adaptive statistical reconstruction (ASiR). Following exploratory phantom work, 33 adult patients of various sizes were scanned using the Siemens scanner and matched to patients scanned using our established GE protocol to give 33 patient pairs. A comparison of volumetric CT dose index (CTDI_vol_) and image noise within these patient pairs informed optimisation, specifically for obese patients. Another matched patient study containing 27 patient pairs was used to confirm protocol matching. Size-specific dose estimates (SSDEs) were calculated for patients in the second cohort. With the acquisition protocol for the Siemens scanner determined, clinicians visually graded the images to identify optimal reconstruction parameters.

**Results:**

In the first matched patient study, the mean percentage difference in CTDI_vol_ for Siemens compared to GE was − 10.7% (range − 41.7 to 50.1%), and the mean percentage difference in noise measured in the patients’ liver was 7.6% (range − 31.0 to 76.8%). In the second matched patient study, the mean percentage difference in CTDI_vol_ for Siemens compared to GE was − 20.5% (range − 43.1 to 1.9%), and the mean percentage difference in noise was 19.8% (range − 27.0 to 146.8%). For these patients, the mean SSDEs for patients scanned on the Siemens and GE scanners were 3.27 (range 2.83 to 4.22) mGy and 4.09 (range 2.81 to 4.82) mGy, respectively. The analysis of the visual grading study indicated no preference for any of the SAFIRE strengths.

**Conclusions:**

Given the different implementations of acquisition parameters and reconstruction algorithms between vendors, careful consideration is required to ensure optimisation and standardisation of protocols.

## Background

Hybrid imaging in the form of positron emission tomography (PET) combined with computed tomography (CT) is a clinical standard in oncology, neurology and cardiology. The CT portion of the study is essential for attenuation correction of the PET data and localisation of the PET tracer uptake within the patient’s body.

Under the Ionising Radiation (Medical Exposure) Regulations, there is a clear obligation to optimise the radiation dose received by patients undergoing any clinical exposure to ionising radiation: to quote the regulations directly, we “must ensure that doses arising from the exposure are kept as low as reasonably practicable” (the ALARP principle) [1]. In PET-CT, the CT portion of the scan could account for up to half of the total radiation dose from the study depending on the injected activity and the scan length. Thus, there is a significant value in ensuring the CT acquisition parameters are optimised to provide the minimum possible radiation dose to the patient with adequate image quality.

Efforts towards optimisation in CT are based on the well-established relationship between image quality and patient radiation exposure. A CT image acquired at a higher dose level is likely to have superior image quality: it will have a higher contrast to noise ratio and will contain fewer artefacts than an image acquired at a lower dose level. What is considered adequate image quality depends directly on how the CT scan is to be utilised. Traditionally, the CT portion of a PET-CT scan is used for attenuation correction, for which comparatively high levels of image noise are acceptable, and localisation of the PET uptake within the patient’s anatomy, for which moderately good image quality is required. It is possible to generate multiple reconstructions of the same CT acquisition to fulfil these different requirements: a smoothed reconstruction can be generated for the attenuation of the PET image, and another reconstruction can be generated for localisation, for example. Whether or not a CT acquisition is to be used for multiple reconstructions, its intended purpose has a significant effect on the appropriate dose level and must inform optimisation [2]. For instance, it is unusual for the CT portion of a PET-CT scan to be read as a fully diagnostic CT; therefore, the patient dose can be much lower than the dose delivered by a stand-alone CT scanner used in a radiology department. This is reflected in the recently published national diagnostic reference levels for hybrid PET-CT and SPECT-CT based on the work by Iball et al. [3], which are considerably lower than the national diagnostic reference levels for diagnostic CT studies [4].

The concept of automatically modulating the X-ray tube current to optimise patient dose in CT originates from the early 1980s [5]. Since then, automatic exposure control (AEC), where the CT acquisition parameters are algorithmically varied by the scanner software to account for the size and shape of the patient, has become widely used in both hybrid imaging and diagnostic CT. The appeal of AEC algorithms is that they automatically modify the dose delivered to the patient depending on the size of the patient. This is achieved by varying the CT tube current (mA) during the scan acquisition in response to variations in the degree of attenuation by different parts of the patient’s anatomy. In general, there are two techniques used, often simultaneously: *z*-axis modulation, where the tube current is varied along the length of the patient, and angular modulation, where the tube current is varied as the tube moves around the patient [6–8]. Radiation dose savings ranging from approximately 15 to 60% have been reported in the literature [9–15], indicating that the efficacy of AEC as a dose reduction technique is highly dependent on the type of algorithm used and the clinical indication.

Each scanner manufacturer has implemented its own AEC algorithm, and the important differences between these algorithms are comprehensively described by several authors [7, 9, 12, 16–19]. The offerings by Siemens and Philips are based on a reference image concept, where the AEC algorithm varies the exposure (measured in milliampere-seconds, or mAs) to provide acceptable image quality, as specified by the operator. For the Siemens algorithm, Care Dose4D [20], the operator selects a *Quality Reference mAs* equal to the mAs that would be required to give acceptable image quality for a 75-kg adult patient. In addition, the operator selects the *strength* of the modulation from five options—very weak, weak, average, strong and very strong—where selecting a strong modulation will result in a larger increase in mAs for larger patients and a larger decrease in mAs for smaller patients, and selecting a weak modulation will result in a smaller increase or decrease.

In contrast, the AEC algorithms offered by General Electric (GE) and Toshiba require the operator to define an acceptable level of noise in the final image. In the GE algorithm, AutomA, the operator defines a *Noise Index* which is used as a surrogate for the target level of noise in the image: the algorithm varies the mAs such that if a uniform phantom was scanned in place of the patient, the standard deviation in the centre of the image would be equal to the Noise Index. A disadvantage of this approach is that it does not take into account the fact that the presence of fat around the organs of larger patients improves inherent contrast; therefore, acceptable image quality can be achieved with higher noise levels compared to smaller patients [16, 21].

The current work focuses on the use of AEC algorithms for adult patients. The use of AEC algorithms for paediatric patients, where optimisation of a dose is paramount due to the increased sensitivity of children to radiation, is clearly also attractive. Optimisation of CT radiation dose is more challenging for paediatrics compared to adult imaging due to the large range of patient sizes. The image quality requirements in paediatric imaging are also somewhat less forgiving owing to the reduced soft tissue contrast and the presence of less adipose tissue between organs [22]. Nevertheless, techniques for protocol optimisation in paediatrics are broadly similar to those for adult protocols [23], and careful implementation of AEC algorithms, based on the adaptation of well-optimised adult protocols, is recommended [22, 24, 25].

In addition to the differences in AEC implementations, scanner manufacturers also offer different reconstruction algorithms. For instance, the ASiR [26] and SAFIRE [27] reconstruction algorithms available on our GE and Siemens scanners, respectively, are both examples of proprietary hybrid iterative reconstruction algorithms. In these algorithms, analytical and iterative methods are combined to produce images with improved image quality compared to images that could be expected from traditional filtered back projection (FBP) [28]. Both vendors’ algorithms allow adjustment of the level of blending of the FBP and iterative portions of their reconstruction algorithm. By adjusting the level of blending, the operator has some control over the level of noise in the final image. In the GE ASiR algorithm, this is achieved via a percentage ASiR blending set by the operator; 40% ASiR blending is used at our centre based on previous work [29]. In the Siemens SAFIRE algorithm, the operator selects one of five *SAFIRE strengths*, where a strength of 1 provides noisier images and a strength of 5 provides smoother images.

Our centre recently commissioned a new scanning suite containing a Siemens Biograph mCT Flow 64 PET-CT scanner with 64-slice SOMATOM Definition AS CT which was purchased predominantly for routine clinical scanning. This was our third PET-CT scanner; the existing scanners were identical GE Discovery 710 scanners with 128-slice Optima 660 CT installed several years earlier and in established clinical use with a suite of CT acquisition protocols. These protocols were established by an optimisation project carried out when the scanners were installed [29], and the clinical experience of the intervening years has provided evidence that the protocols provide adequate image quality for the clinical aims. Furthermore, local dose audits have shown that the doses delivered by the protocols compare favourably with the national diagnostic reference levels [3]. For the purposes of the current study, we therefore considered the established GE protocols to be optimised for clinical purposes. Given the importance of optimisation as well as the desire to standardise our imaging protocols within the department, we were keen to set up protocols on our new Siemens scanner that were matched as closely as possible to the protocols on our existing GE scanners.

Various groups have proposed solutions to the challenge of matching diagnostic CT protocols between standalone CT scanners from different vendors. Both Solomon et al. [30] and Winslow et al. [31] approached the task from the point of view of image quality and proposed methods to characterise and match-specific parameters related to image quality between vendors. McKenney et al. [32] developed quantitative algorithms to translate CT protocols using AEC between GE and Siemens scanners by matching either CTDI_vol_ or image noise. Taking a wider view of CT protocols with a focus on large departments, Szczykutowicz et al. [33] developed a master protocol concept to generate customised CT protocols on scanners from a single vendor but which could be applied to the task of generating protocols across vendors. There is very little in the literature to date regarding the matching of protocols for the CT portion of scans acquired as part of PET-CT studies.

The aim of our study was to address the issue of CT protocol matching between two different PET-CT scanners. We evaluated and optimised a protocol for our new Siemens scanner that was matched to our existing GE scanners. We focussed our efforts on the protocol for half-body scans (from the base of the brain to the mid-thigh), which is used for [^18^F]FDG and [^68^Ga]Ga-DOTA-TATE PET-CT scans at our centre. This is the CT protocol used most frequently for PET-CT at our centre and in most other PET departments.

## Methods

### Preliminary phantom experiment

Acquisition parameters were identified for the Siemens Biograph mCT Flow scanner that would broadly match those of the established half body acquisition protocol on our GE Discovery 710 scanners (hereafter referred to as the GE reference protocol) in terms of patient dose and image quality. A Catphan 500 phantom (The Phantom Laboratory, Salem, NY, USA) was scanned on a GE scanner and on the Siemens scanner. The acquisition parameters used on both scanners are provided in Table 1. The use of a fixed tube voltage of 140 kV on both scanners is based on a desire to minimise errors in PET attenuation correction as quantified by other authors [34] and to ensure adequate image quality for the full range of patient sizes. On the Siemens scanner, a series of scans were acquired with the Quality Reference mAs varied each time from 20 to 60 mAs. The representative tube currents listed in Table 1 are taken from the uniform section of the phantom used for the noise measurements described below.

**Table 1:** CT acquisition parameters used for the preliminary phantom experiment

	GE Discovery 710 (GE reference protocol)	Siemens Biograph mCT Flow
kV	140	140
AEC setting	Noise Index = 40	*Quality Reference mAs varied*
Collimation	64 × 0.625 mm	32 × 0.6 mm
Spiral pitch	1.375	1.35
Reconstructed slice thickness	2.5 mm	2 mm or 3 mm
Reconstructed field of view	500 mm	500 mm
Rotation time	0.5 s	0.5 s
Representative tube current	15 mA	*Varied with Quality Reference mAs (range 20 to 47 mA)*

The protocols on both scanners were set up to generate two CT reconstructions from the single acquisition: one smoothed, large field of view image used by the PET reconstruction algorithm for attenuation of the PET image (the CTAC image) and one for use during clinical reporting. Evaluation of the CTAC image, including checks of the accuracy of the PET attenuation correction, was carried out during the commissioning of the Siemens scanner and is outside the scope of this project. The work described here relates to the images used during clinical reporting.

Image reconstruction was carried out using iterative reconstruction on both scanners: for GE data, the ASiR (Adaptive Statistical Iterative Reconstruction) algorithm [26] was used with 40% blending (as per the GE reference protocol), and for Siemens data, the SAFIRE (Sinogram Affirmed Iterative Reconstruction) algorithm [27] was used with a strength setting of 3 and a kernel of I30f. These SAFIRE settings were recommended by Siemens and used by other centres accredited by the UK PET Core Lab [35]. Since the slice thickness of 2.5 mm in the reference protocol could not be matched exactly on the Siemens scanner, images were reconstructed at both 2 mm and 3 mm slice thickness for comparison.

The volume computed tomography dose index (CTDI_vol_) [36] reported in the scanner-generated DICOM dose report was used as a surrogate for patient dose for this preliminary study. Image quality was quantified according to the noise in the reconstructed images, measured by taking the mean of the pixel standard deviations measured in the regions of interest of 5 cm diameter placed in five slices in the uniform section of the Catphan phantom. All image analysis was performed using Hermes Hybrid Viewer (Hermes Medical Solutions, Stockholm, Sweden).

### Comparison of patient scans for acquisition protocol matching

Thirty-three patients were scanned on the Siemens scanner using the protocol established above. The Care Dose4D strength setting remained on the default *average* setting for these scans. Each patient scanned on the Siemens scanner was matched by gender, height, weight and scanning position (arms up above the head or by the side of the body) to a patient scanned on a GE scanner using the GE reference protocol. Each patient’s body mass index (BMI) was calculated as their weight in kilogrammes divided by the square of their height in metres. Each patient pair was categorised according to the ranges defined by the World Health Organization (WHO) [37] as underweight (BMI less than 18.5 kg/m^2^), normal weight (BMI between 18.5 and 25 kg/m^2^), overweight (BMI between 25 and 30 kg/m^2^) or obese (BMI over 30 kg/m^2^). All patient images were reviewed to check for proper centring of the patient inside the field of view, in order to ensure optimal function of the AEC algorithms.

As in the preliminary phantom experiment, the CTDI_vol_ reported in the scanner-generated DICOM dose report was used as a surrogate for the patient dose to compare the matched patient cohorts on the Siemens and GE scanners. Images were reconstructed using the ASiR and SAFIRE reconstruction algorithms described in the previous section. The image quality was then quantified according to the image noise measured as the pixel standard deviation in a 3-cm circular region of interest positioned in a uniform section of the patient’s liver. Plots of CTDI_vol_ and image noise against patient BMI were used to compare how the Siemens and GE protocols performed over the range of patient sizes.

Upon evaluation of the above patient study, we identified two patients scanned on the Siemens scanner for whom the CTDI_vol_ was significantly higher than their matched counterparts scanned on a GE scanner. Both patients were in the obese BMI category. The Care Dose4D strength setting was therefore changed from *average* to *weak* for the adult obese patient group only. A second matched patient study was then carried out using the method described above with a further 27 patient pairs scanned on the Siemens and GE scanners.

Finally, since the CTDI_vol_ does not provide a measure of the radiation dose delivered to the patient (rather, it simply measures the radiation output of the CT scanner), size-specific dose estimates (SSDEs) for the patients included in the second matched patient study were calculated according to the protocol in the American Association of Physicists (AAPM) Report No. 204 [38]. The SSDE is a patient dose estimate that is calculated by multiplying the CTDI_vol_ by a factor calculated from the physical dimensions of the patient’s body, as measured on the CT image. The SSDE is a more appropriate method of estimating and comparing the patient radiation dose delivered by the Siemens and GE protocols as it takes into account patient size.

### Visual grading analysis for evaluation of reconstruction settings

Given the differences in the reconstruction algorithms used by the two vendors, a visual grading study was performed to compare Siemens and GE images in order to identify the optimal SAFIRE strength for our protocol on the new Siemens scanner. Eight patients scanned on the Siemens scanner were selected. This group comprised two patients from each of the four BMI categories (underweight, normal, overweight and obese). Within each BMI category, one patient had been scanned with their arms up above their head, and one patient had been scanned with their arms down by the sides of their body. Each of these patients was matched with a patient scanned using the reference protocol on a GE scanner according to the same criteria used for the matched patient studies described above.

For each patient scanned on the Siemens scanner, three reconstructions of the CT acquisition were generated with the SAFIRE strength set to 2, 3 and 4 (with strengths 1 and 5 having been excluded based on an initial review of the images). The GE images were reconstructed according to the GE reference protocol using ASiR with 40% blending. Whilst blinded to patient details and SAFIRE strength, three clinicians experienced in PET-CT reporting were asked to provide an image quality rating for each Siemens reconstruction on a scale of 1 (unacceptable) to 5 (excellent) for image quality and a comparison rating on a scale of − 2 (Siemens much worse than GE) to + 2 (Siemens much better than GE) to indicate whether they considered the image quality to be better or worse than the matched GE image. The resulting data were analysed to provide visual grading analysis (VGA) [39] scores for each SAFIRE strength, with the VGA score given by the mean score awarded by the clinicians to each group of reconstructions. Visual grading characteristics (VGC) analysis was carried out according to the method proposed by Båth and Månsson [40], with the VGA scores plotted in a manner similar to receiver operating characteristic (ROC) curves to identify any preference for a particular SAFIRE strength.

## Results

### Preliminary phantom experiment

**Fig. 1:**
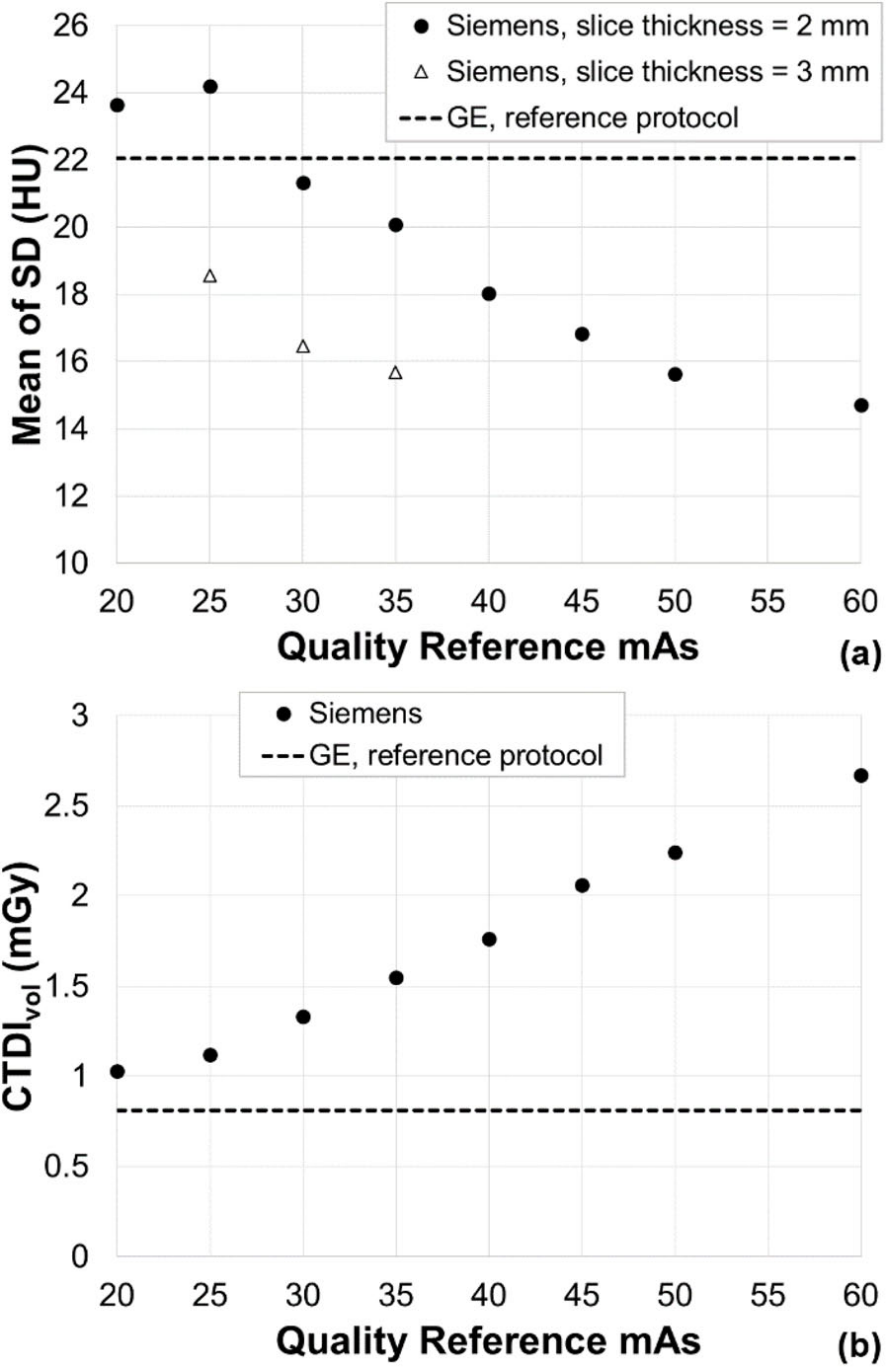
**a** Standard deviation (SD) in a reference region. **b** CTDI_vol_ for the Siemens acquisitions over the range of Quality Reference mAs values. The corresponding values for the reference protocol are also shown

The CTDI_vol_ delivered to the Catphan phantom by the GE reference protocol was 0.81 mGy. The noise (mean pixel standard deviation) in the Catphan phantom images from the GE reference protocol acquisition was 22.05 HU. The corresponding results for the Siemens acquisitions over the range of Quality Reference mAs values are plotted in Fig. 1.

From Fig. 1a, we concluded that the image noise provided by the reference protocol was best matched on the Siemens scanner using a Quality Reference mAs of 30 mAs with a slice thickness of 2 mm. Figure 1b suggests that the CTDI_vol_ level achieved by the reference protocol could not be matched by the new scanner. Using a Quality Reference mAs of 30, mAs delivered a CTDI_vol_ of 1.33 mGy, which is 64% higher than the reference protocol.

Given the importance of maintaining clinical image quality on the Siemens scanner compared to our established GE scanners, a Quality Reference mAs of 30 mAs and a slice thickness of 2 mm were chosen for the Siemens protocol even though the CTDI_vol_ values delivered to the phantom were higher. The intention was to further evaluate and optimise the Siemens protocol based on data from clinical patient scanning.

### Comparison of patient scans for acquisition protocol matching

**Fig. 2:**
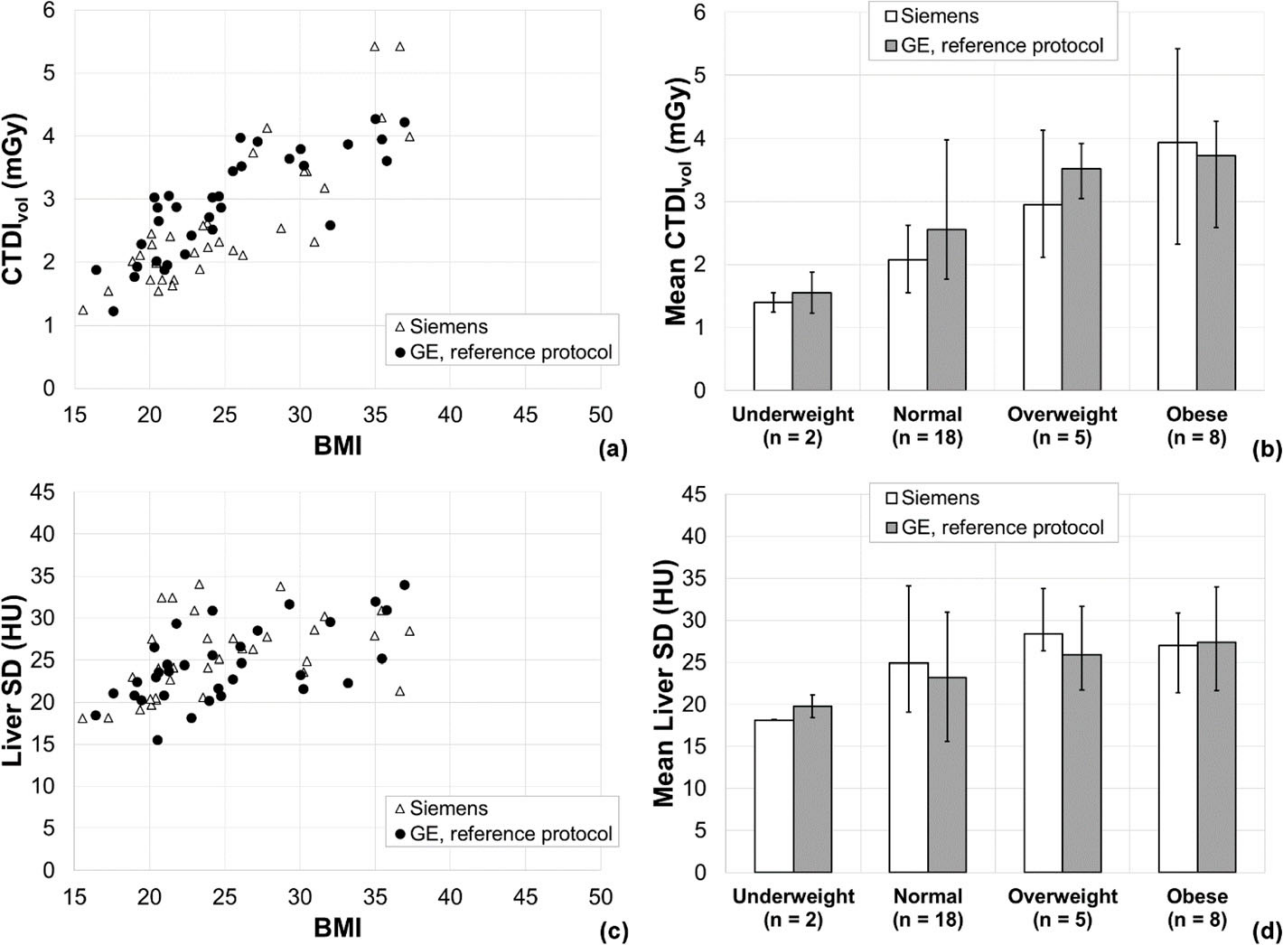
Results of the first matched patient study comparing the new Siemens protocol to the GE reference protocol. **a**, **c** Scatter plots of the two matched patient groups showing a relationship between CTDI_**vol**_ and patient BMI, and liver standard deviation and BMI, respectively. **b**, **d** The same data grouped by BMI category. The error bars indicate the range of each group. The two Siemens patients with a CTDI_**vol**_ above 5 mGy in **a** are the patients that prompted the optimisation of the protocol for the obese patient group

**Fig. 3:**
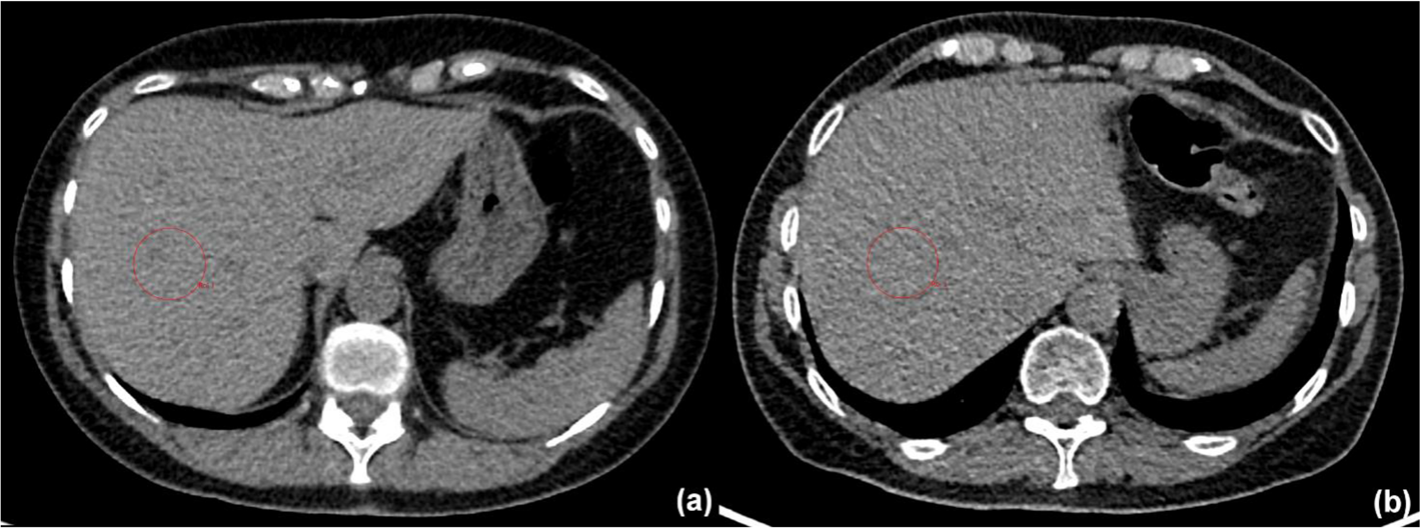
Example matched patient images. **a** Patient (BMI 23.8) scanned with the GE reference protocol; CTDI_vol_ = 2.75 mGy, liver ROI standard deviation = 19.7. **b** Patient (BMI 23.8) scanned on the Siemens scanner; CTDI_vol_ = 2.06 mGy, liver ROI standard deviation = 23.8. The red circles show the regions of interest used for the noise quantification

Figure 2 shows the results of the first matched patient study. The mean percentage difference in CTDI_vol_ for patients scanned on the Siemens scanner compared to the GE reference protocol was − 10.7% (range − 41.7 to 50.1%), and the mean percentage difference in noise was 7.6% (range − 31.0 to 76.8%). In contrast to the phantom results above, the patient doses appeared to be slightly lower overall on the Siemens scanner with an associated slight increase in image noise. Given that the clinicians considered the image quality to be satisfactory, we considered that these results reflected an overall acceptable level of optimisation of the new protocol. As an example of the image quality obtained on the two scanners, Fig. 3 shows images from a pair of matched patients in the normal BMI category.

Despite the overall acceptability of the results from the first matched patient study, some room for optimisation was identified for obese patients; for two out of eight patient pairs, the CTDI_vol_ for the Siemens patients were 37% and 50% higher than for the corresponding GE patients. The Care Dose4D strength setting was changed from *average* to *weak* for the adult obese patient group only, with the expectation that this would reduce the likelihood of the Siemens scanner delivering unacceptably high doses to obese patients.

**Fig. 4:**
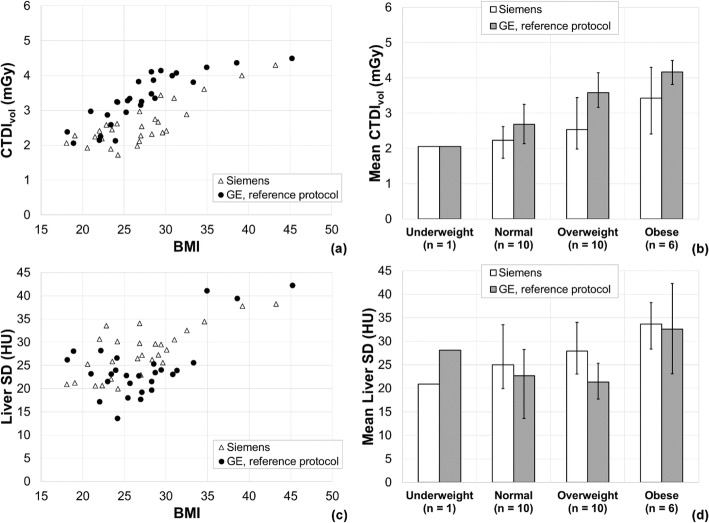
Results of the second matched patient study comparing the new Siemens protocol to the GE reference protocol. **a**, **c** Scatter plots of the two matched patient groups showing a relationship between CTDI_vol_ and patient BMI, and liver standard deviation and BMI, respectively. **b**, **d** The same data grouped by BMI category. The error bars indicate the range of each group

**Fig. 5:**
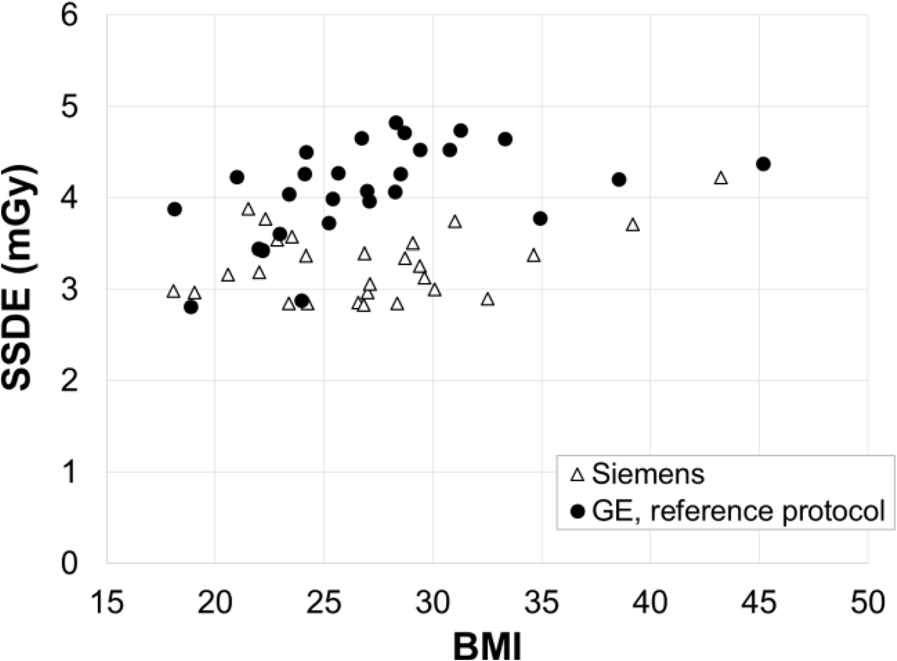
Scatter plot of size-specific dose estimates (SSDEs) against BMI for patients in the second matched patient study

The results of the second matched patient study, which was carried out after changing the Care Dose4D strength setting, are shown in Fig. 4. Figure 5 shows the SSDEs calculated for the second matched patient study. The mean SSDEs for the patients scanned on the Siemens scanner and with the GE reference protocol were 3.27 (range 2.83 to 4.22) mGy and 4.09 (range 2.81 to 4.82) mGy, respectively. It is notable that the CTDI_vol_ delivered by both vendors is seen to increase with patient size (see Figs. 2a and 4a), but the SSDE is much less variable with patient size (see Fig. 5). This indicates that the radiation dose received by patients being scanned using either the Siemens or GE protocol is, broadly speaking, independent of their size.

### Visual grading analysis for evaluation of reconstruction settings

**Fig. 6:**
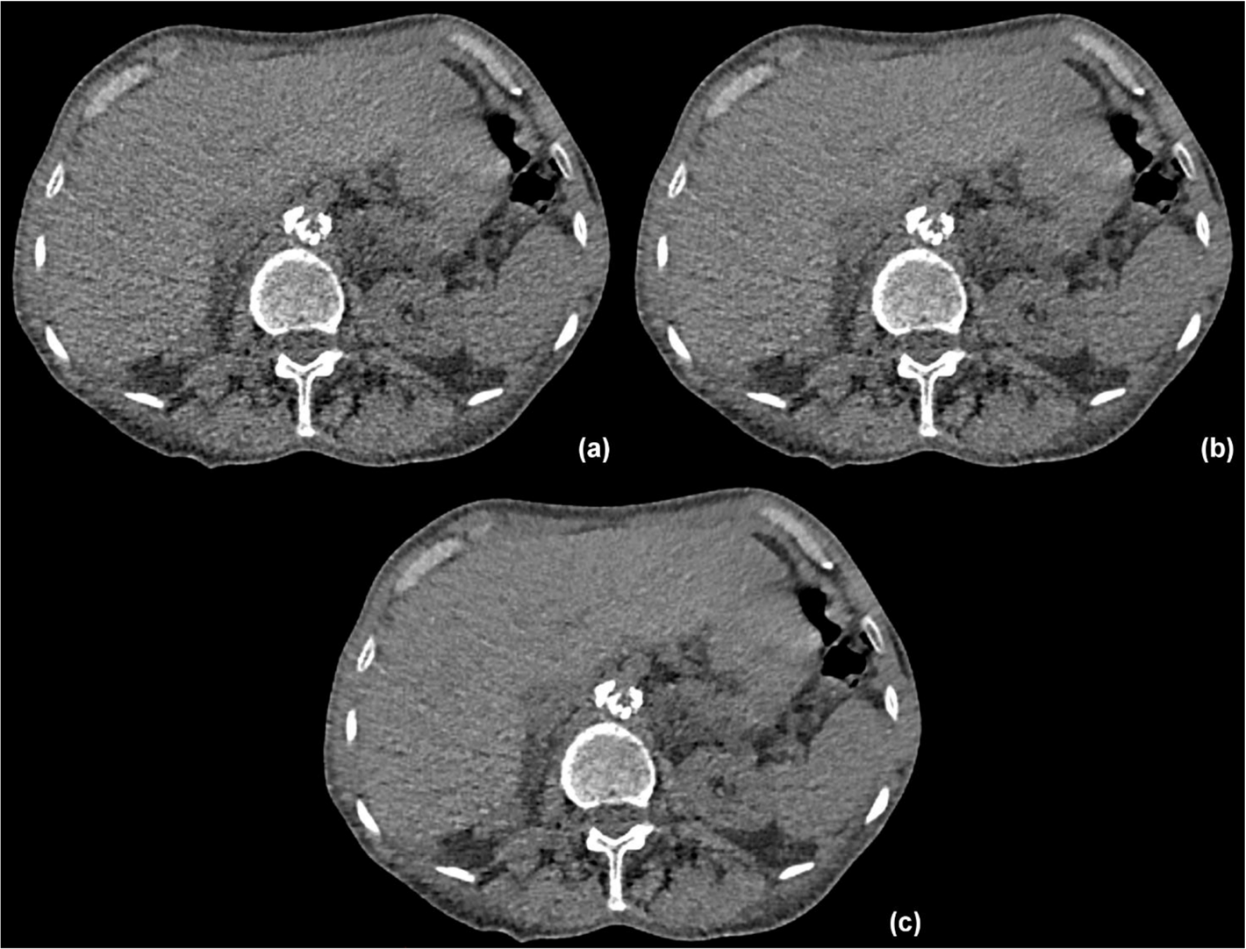
**a**–**c** The same image reconstructed with the SAFIRE strength set to 2, 3 and 4, respectively. Standard deviations measured in the patient’s liver in these images were 28.1, 23.2 and 20.8 for **a**, **b** and **c**, respectively

Figure 6 shows the effect of changing the SAFIRE strength on the appearance of the final image. For this patient, the image noise (measured as the standard deviation in a 3-cm circular region of interest placed in the patient’s liver) for SAFIRE strengths 2, 3 and 4 were 28.1, 23.2 and 20.8, respectively: increasing the SAFIRE strength is seen to provide smoother images with reduced noise.

Table 2 shows the VGA scores obtained from the visual grading study. The image quality rating VGA scores indicate that the clinicians were satisfied with the image quality for each of the three SAFIRE strengths. The comparison rating VGA scores indicate a slight preference for the images from the Siemens scanner compared to the images from the GE scanner.

**Table 2:** VGA scores for the clinician scoring study

SAFIRE strength	VGA score	
	Image quality rating	Comparison rating
2	3.57	0.25
3	3.75	0.21
4	3.55	0.17

**Fig. 7:**
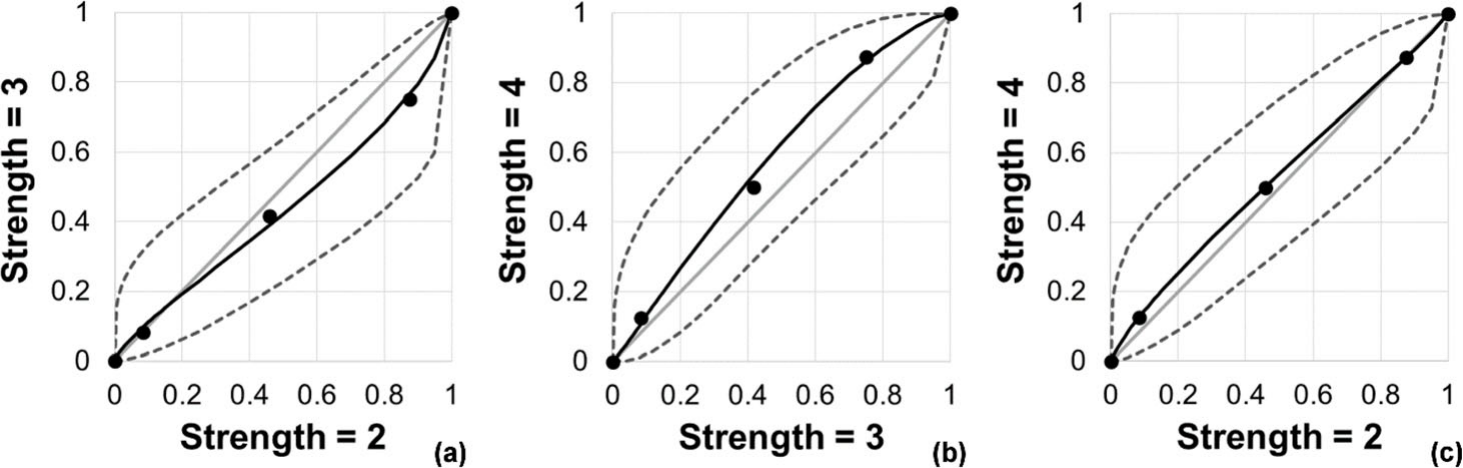
Visual grading characteristic curves comparing each combination of SAFIRE strengths. **a** Strengths 3 vs 2. **b** Strengths 4 vs 3. **c** Strengths 4 vs 2. The dashed lines indicate the 95% confidence intervals. For identically scored images, the curve would fall along the grey line of equality shown in the graphs

Figure 7 shows VGC curves generated from the image quality rating VGA scores. The data used to plot these curves were generated using a web-based calculator for ROC curves developed by Eng and Morgan [41]. The VGC curves do not significantly deviate from the line of equality for any combination of SAFIRE strengths, indicating that the clinician scoring study did not reveal a preference for any SAFIRE strength. Given this result, the decision was made to continue using the default SAFIRE strength of 3 for half-body scanning on the new Siemens scanner.

## Discussion

Our aim was to generate and evaluate a low-dose CT protocol on our new Siemens scanner that was satisfactorily matched to our existing GE clinical protocol. We used a multi-step approach to achieve this aim, starting with a phantom experiment to broadly identify appropriate acquisition parameters on the new scanner and then using patient studies to evaluate and fine-tune the acquisition and reconstruction parameters. The resultant Siemens protocol provides satisfactory image quality at a slightly lower average patient dose compared to the GE protocol.

The use of the Catphan 500 phantom for the preliminary assessment was driven by the availability of this phantom at our centre. This phantom is significantly smaller in diameter than a patient. We believe this explains the discrepancy seen between the results of the phantom experiment and the patient comparisons that followed, namely that the CTDI_vol_ values were higher for the Siemens acquisitions than the GE acquisitions for the phantom and lower on average for patients. However, we believe that despite this limitation, the phantom experiment gave us valuable data to broadly compare the dose levels delivered by the two scanners and provided a reasonable starting point for our patient optimisation study. Indeed, the phantom experiment provided the Quality Reference mAs value of 30 mAs which we found to be appropriate for patient scans.

The task of protocol matching was complicated by the fact that the two vendors have AEC implementations that are based on different approaches (standardisation of noise for GE and standardisation of dose for Siemens). The different implementations are evident in the differing shapes of the relationship between CTDI_vol_ and patient size for the two vendors seen in Figs. 2a and 4a and the cause of the large ranges in the percentage differences calculated in the matched patient studies. On average, the two scanners provide similar patient dose and image noise, but for individual patients, we observed relatively large differences in patient dose and image noise. These differences are unavoidable when using scanners from different vendors.

One important difference between the two vendors is that the GE scanner has a user-defined maximum mA whereas the Siemens scanner does not allow the user to specify a maximum tube output. This is the cause of the comparatively high doses delivered to two of the Siemens patients in the obese category; for their counterparts on the GE scanner, the maximum mA (100 mA) was reached and the patient dose was limited at the expense of poorer image quality. Which of these outcomes—limited patient dose or potentially sub-optimal image quality—is preferable is a matter of continued debate.

The power of the visual grading study to evaluate the three SAFIRE strengths is limited by the relatively low number of patients included in the study (8 patients from each scanner and a total of 32 reconstructions). This number of patients was chosen to ensure the inclusion of patients across the full range of sizes scanned with their arms up above the head or by the side of the body. We believe that this approach was commensurate with the practical aims of the study to provide an optimised protocol for the Siemens scanner well-matched to the GE protocol. The lack of preference for any SAFIRE strength in the visual grading study is likely to be due to the similarity between the three reconstructions, which have only slight differences in terms of their noise properties. The choice of SAFIRE strength is specific to the imaging task, and further evaluation will be required for other CT applications. For instance, in cases where a CT reconstruction is to be used for the visualisation of relatively small malignant lesions, it would be essential to characterise and evaluate the reconstruction algorithm using more in-depth assessment metrics such as the quantitative assessment of contrast-to-noise ratio and resolution.

We used a simple metric—the standard deviation in a single region of interest—as the sole quantitative evaluator of image quality in the phantom study and matched patient studies. This method does not account for other important aspects of image quality such as resolution or contrast-to-noise ratio and is at best a broad indication of image quality. The visual grading study is another imperfect measure of image quality given that it relies on the subjective opinions of clinicians, albeit those with substantial reporting experience. However, we consider that the combination of the two methods of image quality evaluation is sufficient to match protocols between scanners.

Useful further work would extend this study to generate AEC protocols for paediatric imaging. The application of AEC techniques to paediatrics has been investigated somewhat less extensively compared to adult imaging, and there is very little in the literature with regard to the use of AEC for paediatric PET-CT imaging or for the matching of protocols between scanners. In diagnostic imaging of the thorax and abdomen, Greess et al. [42] demonstrated reductions of 26 to 43% using Care Dose4D in paediatric patients. In two studies [15, 43] using anthropomorphic phantoms, Papadakis et al. found dose reduction using Care Dose4D to be inferior in paediatrics compared to adults and that the algorithm sometimes delivered increased doses to child-sized phantoms. It should be noted, however, that these studies made use of a previous version of the Care Dose4D algorithm with separate reference patients for children and adults. More recently, Greffier et al. [44] used Care Dose4D to obtain dose reductions of 43 to 91% for anthropomorphic phantoms representing paediatric patients and used iterative reconstruction to maintain constant image quality indices, suggesting the opportunity for significant optimisation compared to fixed-dose techniques.

Given that we were required only to transfer a single protocol to one new scanner, our approach to protocol matching was deliberately less in-depth than work presented by others in the literature [30–33]. We found that a simple phantom experiment followed by a matched patient study containing a moderate number of patients was adequate to devise and evaluate the new protocol. We believe that this is a practical, workable solution for colleagues in other centres facing the same task.

## Conclusions

Careful choice of parameters used for automatic exposure control in CT protocols is essential to ensure patient doses are optimised according to the ALARP principle. Standardisation of protocols between scanners is an important tool for optimisation and is necessary to allow clinicians to report scans acquired on scanners from multiple vendors. We hope that the methodology we have presented will be helpful to other centres as a simple, practical solution to the challenge of matching protocols across scanner vendors.

## Data Availability

The datasets used and analysed during the current study are available from the corresponding author on reasonable request.
